# IgH 3’ regulatory region increases ectopic class switch recombination

**DOI:** 10.1371/journal.pgen.1009288

**Published:** 2021-02-08

**Authors:** Sandrine Le Noir, Amélie Bonaud, Bastien Hervé, Audrey Baylet, François Boyer, Sandrine Lecardeur, Zeliha Oruc, Christophe Sirac, Michel Cogné

**Affiliations:** 1 UMR CNRS 7276, INSERM 1262 and Université de Limoges: Contrôle de la Réponse Immune B et Lymphoprolifération, 2 rue du Pr. Descottes, Limoges, France; 2 Université de Limoges, US 42/UMS2015 plateforme Biologie Intégrative Santé Chimie Environnement (BISCEm), Limoges, France; 3 Institut Universitaire de France, Paris, France; University of Toronto, CANADA

## Abstract

DNA lesions inflicted by activation-induced deaminase (AID) instrumentally initiate the processes reshaping immunoglobulin genes in mature B-cells, from local somatic hypermutation (SHM) to junctions of distant breaks during class switch recombination (CSR). It remains incompletely understood how these divergent outcomes of AID attacks are differentially and temporally focused, with CSR strictly occurring in the Ig heavy chain (*IgH*) locus while SHM concentrates on rearranged V(D)J regions in the *IgH* and Ig light chain loci. In the *IgH* locus, disruption of either the 3’Regulatory Region (3’RR) super-enhancer or of switch (S) regions preceding constant genes, profoundly affects CSR. Reciprocally, we now examined if these elements are sufficient to induce CSR in a synthetic locus based on the *Igκ* locus backbone. Addition of a surrogate “core 3’RR” (c3’RR) and of a pair of transcribed and spliced Switch regions, together with a reporter system for “κ-CSR” yielded a switchable *Igκ* locus. While the c3’RR stimulated SHM at S regions, it also lowered the local SHM threshold necessary for switch recombination to occur. The 3’RR thus both helps recruit AID to initiate DNA lesions, but then also promotes their resolution through long-distance synapses and recombination following double-strand breaks.

## Author summary

Class switching allows B lymphocytes to replace expression of immunoglobin M with that of immunoglobulins G, A or E. The genetic support of class switching, is a unique and large deletion uniquely occuring within the immunoglobulin heavy chain (IgH) locus. This recombination is triggered after DNA lesions inflicted by the activation-induced deaminase (AID) enzyme. In immunoglobulin light chain loci, AID only stimulates somatic hypermutation. In such a non-IgH locus, we now show that the IgH 3’ superenhancer can promote junctions between distant DNA breaks and ectopic class switch recombination. This study identifies the minimal elements necessary for class-switch recombination to occur instead of hypermutation in a locus targeted by AID, *i*.*e*. transcribed (and spliced) target sites for AID in so-called S regions, and the 3’IgH superenhancer which both helps recruit AID for DNA lesions, and helps repair these lesions through distant gene synapsis and recombination.

## Introduction

The Activation-Induced Deaminase (AID) enzyme has multiple roles in the B-cell lineage and their differential regulation remains to be fully characterized. In all species synthesizing Ig, AID primarily provides Ig variable (V) gene diversification through SHM or gene conversion (GCV) [1]. This ancestral repertoire broadening is even shared with an AID ortholog for the lamprey antigen receptors [2]. In frogs, birds and mammals, evolution endowed AID with the additional role to initiate CSR after DNA lesions affecting target S regions upstream of *IgH* constant (*C*_*H*_) genes [3].

In mouse B-cells, the 3’Regulatory Region super-enhancer (3’RR) is the master *cis-*regulatory element controlling the activities of AID within the *IgH* locus, either for SHM, CSR or locus suicide recombination (LSR) [4–7]. SHM and CSR follow B-cell activation. AID lesions in S regions initiate some low-level local SHM but more dramatically yield DNA double strand breaks (DSBs), followed by junctions at long distances [8]. The 3’RR modulates germline transcription of *C*_*H*_ genes in activated B-cells, chromatin remodeling of S regions and AID recruitment to acceptor S regions [9]. Mammalian S regions consist of 1–10 kb-long highly repetitive G-rich DNA sequences containing clustered RGYW AID consensus motifs [10]. The mouse *3’RR* includes 4 core enhancers: *hs3a*, *hs1*,*2*, *hs3b* and *hs4*. In addition to binding specific transcription factors, enhancers included in the 3’RR are transcribed into eRNA [7], and their function is regulated by a distantly transcribed long non-coding RNA (lncRNA) [11]. The first three enhancers are embedded within a ~25-kb dyad symmetry, while the fourth stands downstream [12, 13]. The four core enhancers combined into a short (2.1 kb) “core 3’RR” (c3’RR) show strong synergy and transcriptional activity, although not reaching that of the complete 30kb-long full-length *3’RR* [13].

The specific contribution of the *3’RR* in SHM, DNA breakage and joining of broken S regions remains elusive, since multiple knock-out experiments (KO) in the *IgH* locus all showed global defects and failed to uncouple the 3’RR functions in transcription or initiation of SHM and CSR [14]. While the 3’RR clearly promotes SHM and carries at least a dual role regarding both SHM and CSR, its influence on CSR is the best documented and most critical in the *IgH* locus. It is necessary for optimal germline transcription of acceptor S regions and its deletion almost abrogates accessibility of these S regions while preserving some accessibility to Sμ [4–6, 15]. S regions from 3’RR-deficient B-cells also show a loss of the chromatin marks H3K9ac and H3K4me3, for which they are normally enriched prior to CSR in activated B-cells [15–18]. Finally, the 3’RR likely helps synapsis of broken S regions, then defining chromosomal loops between S regions that stand on the same allele [19].

While the 3’RR helps recruit AID, it thus clearly mediates additional effects that are important for CSR. An ideal way to evaluate those additional effects is to introduce the c3’RR into a locus that efficiently recruits AID beforehand for SHM.

In a previously developed model of ectopic CSR, a complete CSR substrate was designed, providing all the S sequences, transcription and splicing patterns known to be important for local recruitment of AID [20]. This substrate was introduced into the *Igκ* “*KIKS”* locus and underwent high transcription accompanied by efficient SHM and presence of localized internal S region deletions (ISD) [20]. Despite this efficient targeting by AID, “κ-CSR” events joining a pair of ectopic S regions remained exceptional and clearly much rarer than for “IgH-CSR”, with a defect likely affecting the occurrence of synapses between distant AID targets [20].

We hypothesized that these ectopic S regions inserted into an *Igκ* location exposed to AID, would provide an ideal model to explore the role of the 3’RR beyond AID recruitment and check whether this super-enhancer could be the missing piece of an *Igκ* switchable locus. We thus tried complementing the *KIKS* CSR substrate with a core “*c3’RR*” cassette, in order to facilitate tethering of two distant κ-S regions and check whether this could raise the level of κ-CSR closer to that of classical IgH-CSR.

## Material and methods

### Ethics statement

Procedures were reviewed and approved by the Ministère de l’Education Nationale de l’Enseignement Supérieur et Recherche autorisation APAFIS#16151-2018071716292105v3.

### Mice

The strategy used to generate *c3’RR-KIKS* mice was identical to that previously described for *KIKS* mice [20], except for the inclusion of a c3’RR cassette downstream of the genomic fragment containing the *Eiκ* enhancer and *Cκ* gene. The *c3’RR* cassette included all four *3’RR* enhancers in their normal palindromic layout, as described [13]. Knock-in was done in CK35 embryonic stem cells, which were injected into blastocysts. After germline transmission, mice were bred with a *cre-*expressing strain to delete the Neo^R^ cassette. *KIKS*, *c3’RR-KIKS* and *c3’RR-KIKS* / *Aicda*^*-/-*^ mice were used. All animal strains were bred and maintained in SOPF conditions.

### ELISA assays

ELISAs for the presence of murine and human Igκ were performed on serum from *KIKS*, *c3’RR-KIKS* and *c3’RR-KIKS* / *Aicda*^*-/-*^ as described [20].

### Flow cytometry and cell sorting

Antibodies used for staining and sorting are detailed in S1 Table. Flow cytometry analyses were done on a BD Pharmingen LSRFortessa cytometer. Data were then analyzed with BD FACSDiva software (BD Biosciences, San Jose, CA). Gating strategy is shown in S2 Fig. Sorting was performed on a FACS ARIA 3 (BD Biosciences).

### Immunization

Groups of 8-week-old mice were immunized by intraperitoneal injection of 200 μl SRBC and analyzed 8 days later.

### SHM and switch junction analyses

SHM and switch junction analyses were performed on B220^+^/GL7^+^ and B220^+^/Igκ^+^ cells from Peyer’s patches, respectively, using previously described primers for *Jκ5* 3’ flanking intron and switch junction [20] and LVk4-63F: 5'- TGC AGA TTT TCA GCT TCC TG-3' and Sγ3R: 5'- CCT CAC CCA CCC TAG CTC A-3' for κ-Sγ3 region. PCR products (100ng) were fragmented using Ion Shear Plus reagents kit (Life Technologies), then barcodes and adaptors were ligated using Ion Xpress Plus Fragment (Life Technologies). Fragments around 200pb were selected using E-Gel Size Select 2% (Life Technologies) and sequenced on an Ion Proton System. Raw files were generated using Ion Torrent Suite (adapter- and barcode-trimmed), and junctions were analyzed using CSReport, together with mutation frequency [21]. Mutation frequency was analyzed using DeMinEr tool [22]. Sequences are deposited under the reference PRJEB34851.

### Transcript analysis

Total RNA from Peyer’s patches was extracted with TRI Reagent (Ambion, Austin, TX), and 1 μg was used for cDNA synthesis using random primers (Applied Biosystems). Relative quantification of primary transcripts was performed with SYBR green Master Mix (Applied Biosystems) by reference to GADPH levels. QPCR primers and assays were as previously described [20].

### Cell culture

Splenocytes were collected, red blood cells were lysed and cells were CD43-depleted using CD43 microbeads (Miltenyi Biotec). Splenic B lymphocytes were cultured for 3 days in RPMI containing 10% FCS with LPS (1μg/mL, B4 Invivogen) or LPS + IL4 (20ng/mL, Peprotech). Cells and supernatants were recovered for LAM-HTGTS experiments and ELISA.

### LAM-HTGTS

**Fig 1:**
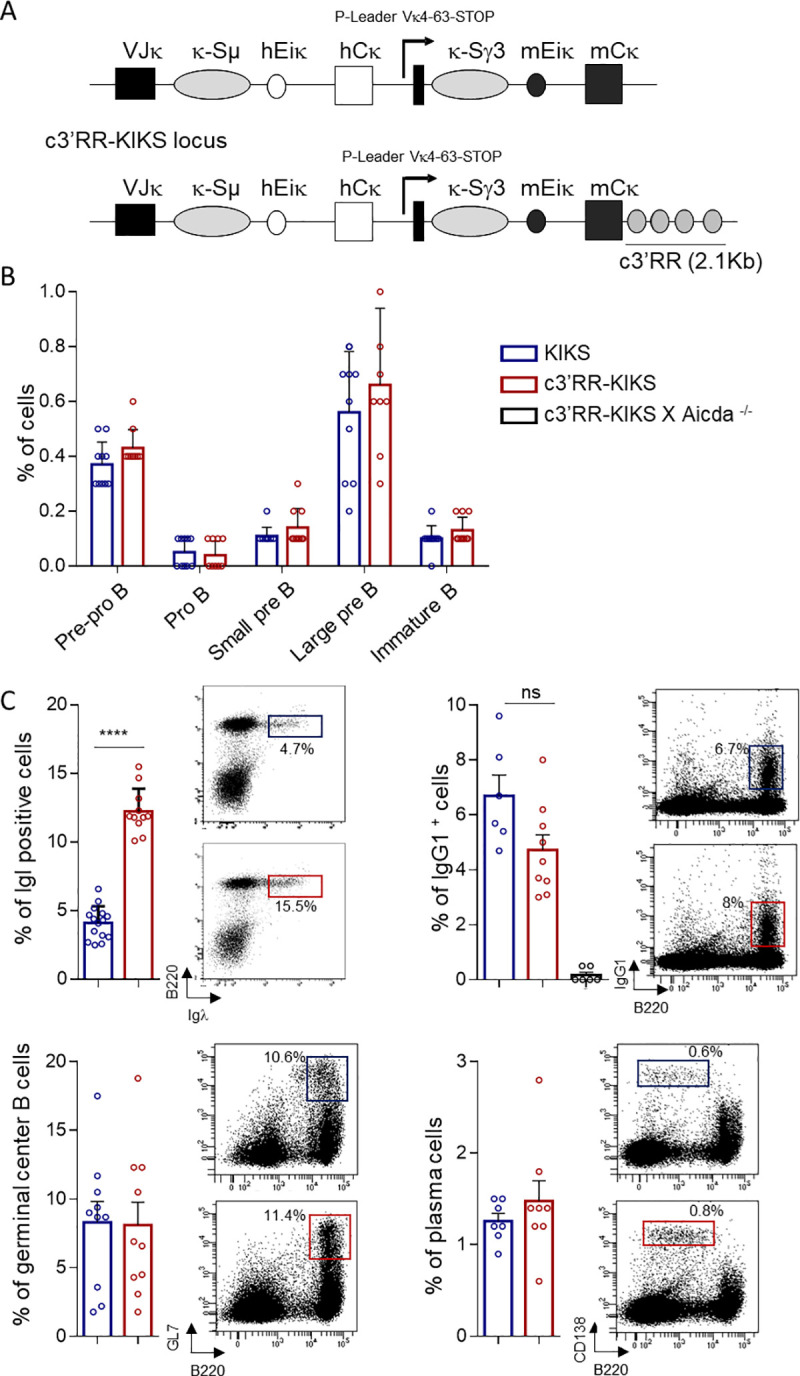
B-cell development. (A) Comparison between switchable Igκ loci. In *c3’RR-KIKS* mice, a *c3’RR* was inserted downstream of the locus. (B) Flow cytometry analysis of B-cell compartments in bone marrow. Percentages are given with mean+/- SEM as determined among total gated bone marrow lymphocytes. Data are representative of 10 animals of each genotype (left panel). (C) Flow cytometry analysis with representative dot plots, in spleen after SRBC immunization (D8), of switched B-cells (B220^+^/IgG1^+^), GC B-cells (B220^+^/GL7^+^) and plasma cells (B220^low^/CD138^+^) and mλ positive cells in non-immunized spleen. For switched B-cells, GC B cells and mλ the % are expressed on B220^+^ cells, and the % of plasma cells are expressed on total lymphocytes. Data are representative of 6 to 10 animals of each genotype. Percentages are given with mean+/- SEM. Mann-Whitney *U*-test for significance.

LAM-HTGTS was performed as previously described [23] using activated splenocytes or Peyer’s patches cells (enriched with the STEMCELL “B-cell isolation kit”). Jκ5-biotin (5’-TGT GTA AGA CAC AGG TTT TCA TGT) and Jκ5-nested (5’- CAG AAA ATC TTG AGA AAA TGG AGA-3’) primers and Sμ primers [24] were used to generate libraries. Data analysis of MiSeq sequencing reads was performed as previously described [25]. Graphical representation were performed as previously described [26]. All sequence alignments were done with the mouse mm10 genome (or its variant sequence corrected for our *KIKS* changes to the *Igκ* locus (Figs 1 and S1). Sequences are deposited under the reference PRJEB34781.

### Statistical analysis

Statistical tests used in the study are indicated in the figure legends and performed using GraphPad Prism (*p<0.05, **p<0.01, ***p<0.001, ****p<0.0001).

## Results and discussion

### c3’RR-KIKS locus description and B-cell development

To determine whether the *3’RR* super-enhancer could by itself affect the output of AID-initiated single nucleotide lesions and increase the balance of distant recombination *vs* local alteration, we studied *Igκ* loci modified in order to support ectopic CSR. The *KIKS Igκ* variant locus (carrying only paired S regions) is known to show low-levels of κ-CSR junctions after AID-mediated DNA lesions [20]. We compared it to a *c3’RR-KIKS* variant carrying not only an κ-*Sμ/κ-Sγ3* pair but also a surrogate *c3’RR* super-enhancer cassette (Figs 1A and S1). The *c3’RR* included the four core enhancers (*hs3a*, *hs1*.*2*, *hs3a* and *hs4*) of the *IgH 3’RR*. We have previously described that the insertion at the *Igκ* locus in the *KIKS* mice does not disturb early and late B cell development compared to *wt* mice. For this reason we only compared the two KI mouse models [20]. Normal early B-cell development occurred in both homozygous *c3’RR-KIKS* mice and *KIKS* mice, showing normal distribution of bone marrow pre-pro B, proB, large preB, small preB and immature B-cell populations (Figs 1B and S2). We only noticed an occasional increase of Ig lambda usage (Igλ) in some *c3’RR-KIKS* compared to *KIKS* mice, suggesting that LC rearrangements might even be accelerated by the knocked-in *IgH* elements, with Igλ then more frequently superseding the early rearranged *Igκ*. Late B-cell development also occurred normally, with no differences either regarding classical *IgH* locus CSR (IgG1^+^ cells in spleen), nor the accumulation of germinal center (GC) B-cells and plasma cells (Fig 1C).

### *The IgH 3’ super-enhancer cassette increases in vivo and in vitro* ectopic κ-CSR *in c3’RR-KIKS mice*

**Fig 2:**
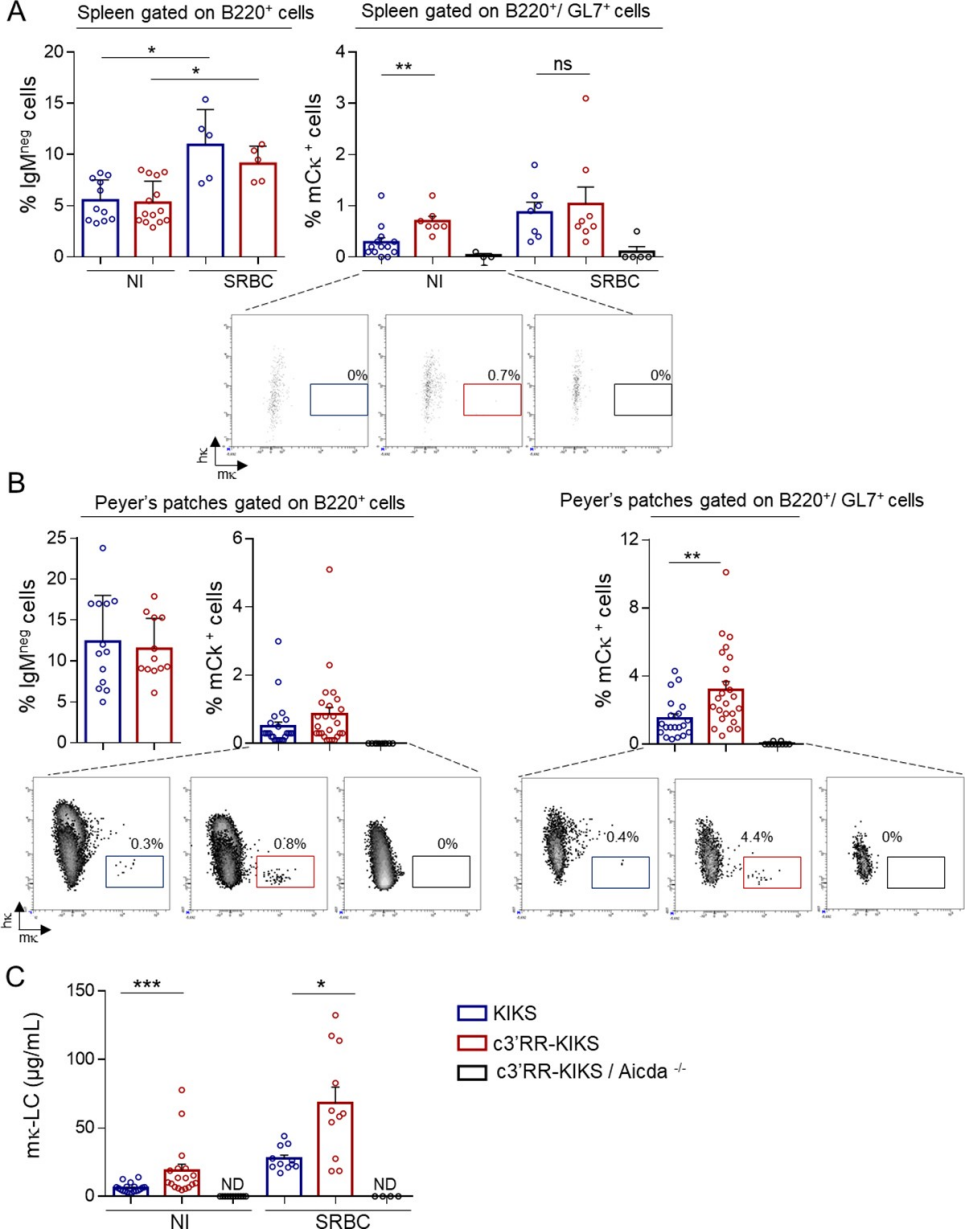
3’RR core enhancers ectopically promote CSR *in vivo*. (A) Left: Evaluation of the amount of cells switching at the *IgH* locus (B220^+^, IgM^neg^ cells) in spleens from NI or SRBC immunized mutant mice (day 8) Right: Comparison of κ-CSR efficiency as evaluated by counting mCκ^+^ spleen B-cells (gated on B220^+^/GL7^+^ cells) by flow cytometry, in spleens from non-immunized (NI) or SRBC immunized (day 8) mice from *KIKS*, *c3’RR-KIKS* and *c3’RR-KIKS / Aicda*^*-/-*^ mice. Percentages +/- SEM are representative of 5 to 13 animals of each genotype (B) Left: Evaluation of the amount of cells switching at the *IgH* locus (B220^+^, IgM^neg^ cells) in Peyer’s patches from *KIKS*, *c3’RR-KIKS* mice. Middle and right: Comparison of κ-CSR efficiency as evaluated by counting mCκ^+^ spleen B-cells either gated on B220 positive cells (*middle*) or on B220^+^/GL7^+^(*right*) by flow cytometry, in Peyer’s patches from *KIKS*, *c3’RR-KIKS* and *c3’RR-KIKS / Aicda*^*-/-*^ mice. Percentages +/- SEM are representative of 10 to 25 animals of each genotype. (C) ELISA quantification of serum mκ-LC in NI and SRBC-immunized mice (Day 8). Percentages +/- SEM are representative of 11 to 19 animals of each genotype.

**Fig 3:**
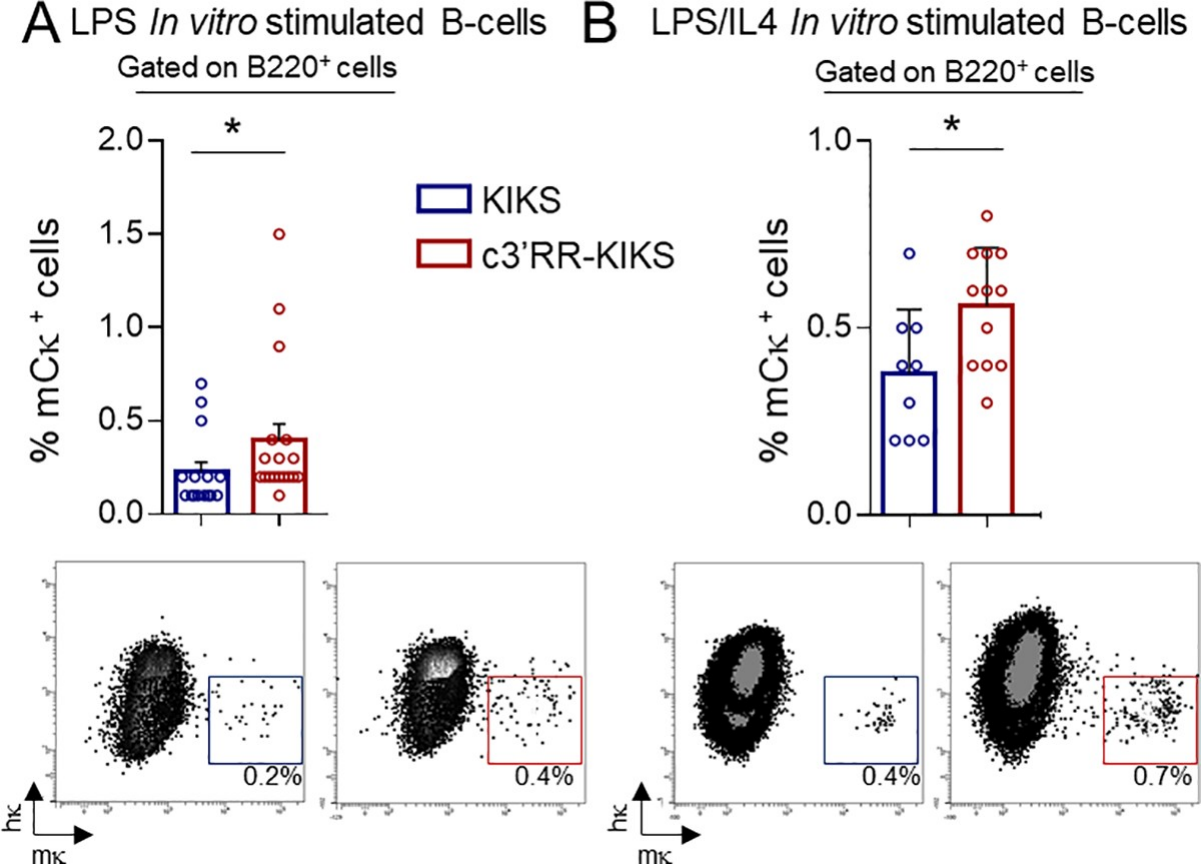
3’RR core enhancers ectopically promote CSR *in vitro*. (A) Comparison of κ-CSR efficiency as evaluated by counting LPS *in vitro* stimulated mCκ^+^ spleen B-cells (gated on B220^+^ cells) by flow cytometry from *KIKS* and *c3’RR-KIKS* mice.(B) as in (A) for LPS/IL4 *in vitro* stimulation. Percentages are given with mean+/- SEM. Mann-Whitney test for significance.

The reporter system included in the *KIKS* and *c3’RR-KIKS* loci was designed in order to monitor κ-CSR loci as a switch from expression of human κ (*hCκ*) to mouse κ-LC (*mCκ*) (Figs 1A and S1). *In vivo*, both *KIKS* and *c3’RR-KIKS* mice developed small numbers of “κ-switched” mCκ-expressing B-cells in spleen. We quantified IgH-switched B cells in spleens from nonimmunized and SRBC-immunized mice by quantifying B220^+^/IgM^-^ cells in which IgG1-switched B cells represented a majority of IgH-switched B cells (Figs 2A and S3A). We first verified that spleen resting B-cells don’t not express mCκ (S3B Fig**).** In nonimmunized spleens, where IgH-switched (B220^+^, IgM^-^) B-cells were only present in low numbers in both models (Fig 2A left panel), κ-CSR was detectable in low amounts and significantly increased under the influence of the *c3’RR* (0.7% in *c3’RR-KIKS vs* 0.28% in *KIKS*, *p = 0*.*0027*) (Fig 2A right panel and S2 Table). After SRBC immunization (boosting classical IgH-CSR in spleens), κ-CSR was also boosted in both models but the trend towards a higher level in *c3’RR-KIKS* mice did not reach statistical significance by comparison to that in *KIKS* mice (Fig 2A right panel and S2 Table). Peyer’s patches represent a chronically inflamed lymphoid tissue, where classical IgH-CSR was higher than in spleens in both models (Fig 2B left panel). κ-CSR was also increased in Peyer’s patches when considering total B-cells (again with a trend to higher κ-CSR in *c3’RR-KIKS vs KIKS* mice but not reaching significance at *p = 0*.*08*) (Fig 2B middle panel and S2 Table). Gated GC B-cells from Peyer’s patches (*i*.*e*. activated GL7^+^ B-cells), showed the clearest difference between both models when focusing on cells where CSR was ongoing, then revealing a significant increase driven by the *c3’RR* (3.2% *vs* 1.5% mCκ^+^ cells, respectively in *c3’RR-KIKS* and *KIKS* mice, p = 0.003) (Fig 2B right panel and S2 Table). The mCκ^+^ cells were detected only in cells that have switched at IgH locus. In both the KIKS and the c3’RR-KIKS mice, we found an almost undetectable level of κCSR (below 0.1%) when the analysis was focused on cells with an unswitched IgH locus (gated as IgM+) (S3C Fig**)**. Although κ-CSR then remained 10 to 15 fold below the parallel IgH-CSR, this confirmed that it could be positively stimulated by integrating an *IgH* super-enhancer cassette into the modified *Igκ* locus. AID-dependence of the κ-CSR process was also confirmed by its abrogation in AID-deficient *c3’RR-KIKS* mice (Fig 2A and 2B). This increased number of mouse Igκ^+^ B-cells at the BCR expression level also translated into a change in amounts of secreted Ig in sera, with secreted mouse κ-LCs significantly increased in both non-immunized (18.8μg/mL *vs* 6.1μg/mL, p = 0.0002) and SRBC-immunized (68μg/mL *vs* 27.6μg/mL, p = 0.016) *c3’RR-KIKS vs KIKS* mice (Fig 2C). We next monitored κ-CSR onset *in vitro* after LPS or LPS/IL4 stimulation. *In vitro* κ-CSR occurred at very low levels but, as *in vivo*, we observed a significant increase (p = 0.033 and p = 0.028 for LPS and LPS/IL4 stimulation, respectively) in mCκ^+^ cells in *c3’RR-KIKS* mice by flow cytometry (Fig 3A and 3B and S2 Table) confirming the role of the c3’RR in κ-CSR regulation.

### Transcription and hypermutation of the knocked-in S regions prior to κ-CSR

**Fig 4:**
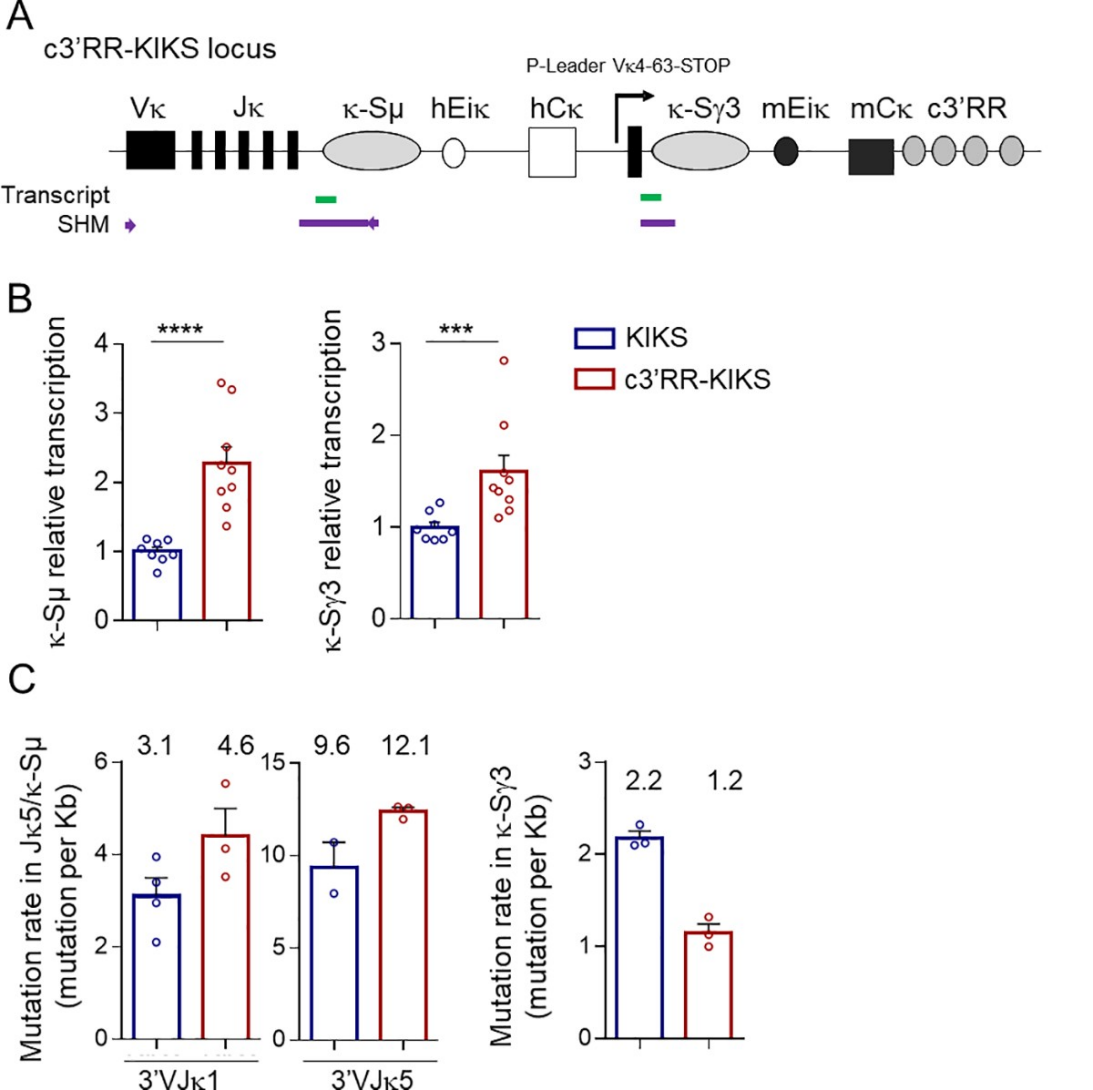
3’RR core enhancers enhance transcription and SHM. (A) c3’RR-KIKS locus with the location of regions (thick lines) that were tested either for transcription (green) or for SHM (purple). Arrows represent primers used for SHM PCR amplification. (B) Relative κ-Sμ and κ-Sγ3 region transcripts analyzed in Peyer’s patch GC B-cells (GC) (n = 8 to 9 mice) from *KIKS* and *c3’RR-KIKS* mice. (C) Mutation rate in the [3’*Jκ5* intron—κ-*Sμ*] region according to the Jκ rearrangement (Jκ1 or Jκ5) (left and middle panels) and mutation rates in the κ-Sγ3 region (right panel) (n = 2 to 4 individual mice) in GC B cells from KIKS, c3’RR-KIKS and *c3’RR-KIKS / Aicda*^*-/-*^ mice. The mutation frequency (mutations per Kb) is indicated over the bar graphs. Data are mean ± SEM, Mann-Whitney test for significance.

AID targeting requires transcription for both SHM and CSR, and we thus assessed the amount of κ-S region primary transcripts in *KIKS* and *c3’RR-KIKS* Peyer’s patches (Fig 4A). Both the κ-Sμ (p<0.0001) and κ-Sγ3 (p = 0.0007) regions proved significantly more transcribed in *c3’RR-KIKS* mice (Fig 4B and S3 Table). As expected upon transcription of a locus that is naturally accessible to AID and before any κ-CSR event, local hypermutation was clearly detectable within unrearranged κ-S regions and within the *Jκ5* 3’ flanking intron from Peyer’s patch GC B-cells. For un-switched κ-*Sμ*, we analyzed 570 bp downstream of *Jκ5*, (*i*.*e*. 220 bp from the 3’ *Jκ5* flanking intron and 350 bp from the inserted κ-*Sμ*). We also analyzed the first 200 bp of un-switched κ-*Sγ3* (Fig 4A). To correct the substitution frequency at each nucleotide position along the sequenced region we used the DeMinEr tool [22], which uses deep sequencing data from mutated (*KIKS* and *c3’RR-KIKS*) and unmutated samples (*c3’RR-KIKS X Aicda* ^*-/-*^). Using NGS, mutation frequencies in the [3’*Jκ5* intron—κ-*Sμ*] region were scored with reference to the *Jκ* rearrangement status (*Jκ1* or *Jκ5*). Confirming previous analyzes in *KIKS* mice [20], a high mutation rate at long distances from the promoter was observed throughout the [3’*Jκ5* intron—κ-*Sμ*] region (Fig 4C and S4 Table). In *c3’RR-KIKS* mice, this mutation rate tended to be higher than in *KIKS* mice (4.6 mut/kb *vs* 3.1 mut/kb for *Jκ1* rearrangement and 12.1 mut/kb *vs* 9.6 mut/kb for *Jκ5* rearrangement) (Fig 4C). By contrast, the mutation load of the acceptor κ-Sγ3 region did not significantly differ (Fig 4C right panel and S4 Table). Thus, inclusion of the *c3’RR* globally increased transcription of κ-S regions and increased SHM around the donor κ-Sμ region in unswitched cells. This confirms the *Igκ* locus as a privileged non-*IgH* location strongly exposing knocked-in S regions to AID, but still with possible modulation by the inclusion of an additional Ig enhancer. Since the increased κ-CSR competence brought by the surrogate *c3’RR* seems to correlate with both higher transcription and with equivalent or higher AID-induced SHM prior to κ-CSR (in *c3’RR-KIKS vs KIKS* mice), we next wanted to precisely compare the structures of recombined switch junctions in both models.

### Molecular analysis of SHM within switch junctions

**Fig 5:**
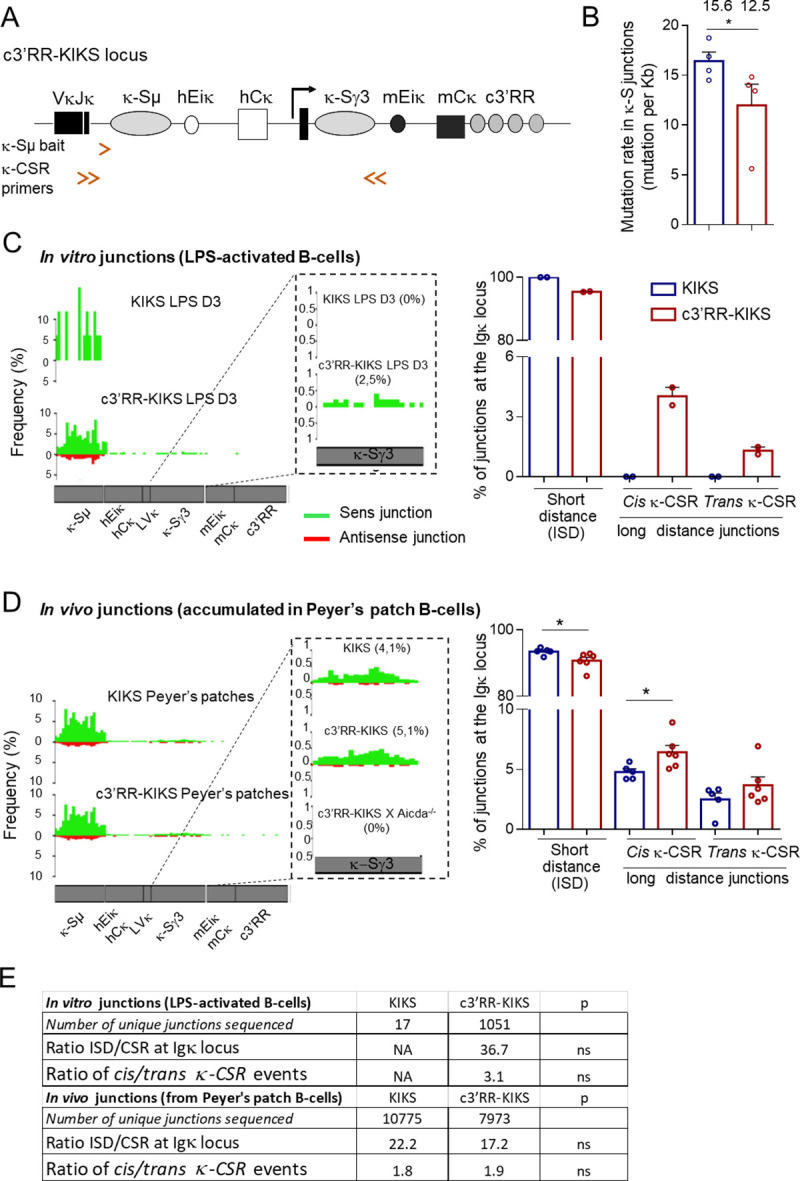
κ-CSR junction analysis. (A) *c3’RR-KIKS* locus with the location of κ-Sμ bait used for LAM-HTGS and primers used for κ-CSR amplification by nested PCR (double arrows). (B) Mutation rates (mutations per kb) in recombined κ-Sμ/Sγ3 junctions are indicated over the bar graphs. κ-Sμ/Sγ3 junctions were obtained by nested-PCR from mCk^+^ cells from Peyer’s patches (n = 4 for each genotype). SHM was analyzed using CSReport. (C) κ-CSR junctions identified by LAM-HTGTS from *in vitro* activated B-cells. Map of junctions identified by LAM-HTGTS along the KI locus (left). Bar graphs (right) with percentages of ISD, *cis* κ-CSR internal to the Igκ locus and *trans* κ*-*CSR (Igκ-IgH) events obtained by LAM-HTGTS. Unique junctional sequences (each corresponding to multiple identical reads) were analyzed; the percentage of “Ig recombination events” (*i*.*e*. junctions of the bait with Ig sequences, either *Igκ* KI or *IgH*) is given. (D) κ-CSR junctions identified by LAM-HTGTS *in vivo* from Peyer’s patch B-cells. (E) Table indicating the number of junctional sequences analyzed, ratio of *cis* / *trans* events, ratio of ISD/CSR within the endogenous *IgH* locus, and ratio of *cis/trans* events.

We analyzed the total mutation load in hybrid κ-S regions from B220^+^/mk^+^ cells from Peyers’ patches by specifically amplifying the rearranged κ-Sμ/κ-Sγ3 regions by nested PCR (Fig 5A). As expected, in both Igκ knock-in configurations, this rate was globally higher for rearranged κ-Sμ/Sγ3 junctions than for unrearranged κ-S regions (Figs 5B and 4C). However, the respective ranking of both models for SHM occurrence then appeared unexpectedly inversed: less SHM was present in rearranged κ-S sequences from *c3’RR-KIKS* (12.5 mut/kb: 93 mutations/7414 pb analyzed) than from *KIKS* B-cells (15.6 mut/kb: 1140 mutations/92472 pb analyzed) (p = 0.029) (Fig 5B). This suggests that in the *Igκ* locus, presence of the c3’RR facilitates / accelerates either the occurrence of DSBs or their repair through synapsis of paired targets. The *3’RR* thus impacts the outcome of AID-initiated lesions and locally tends to increase the recombination *vs* mutation ratio.

To precisely analyze κ-CSR events in our models, we amplified CSR junctions using LAM-HTGTS [23]. By reference to a bait chosen upstream of a given DNA double-stranded break (DSB), this unbiased strategy detects junctions to “preys” located genome-wide. The κ-CSR events were captured by a 3’Jκ5 intron bait (κ-Sμ bait) chosen upstream of the knock-in donor κ-Sμ (Fig 5A). In order to identify and score the sequences joined to baits, non-redundant junctions were then analyzed using the algorithm published by Alt and colleagues [23].

Baits were found to be associated in *cis* or *trans* with sequences from either the *Igκ* KI (*cis* κ- CSR) or the endogenous *IgH* locus (*trans* κ-CSR). Analyzing the partners joined to the 3’Jκ5 intron bait (thereafter “κ-Sμ bait”) in both κ-CSR models, showed that the predominant type of recombination was local intra-Sμ deletion (ISD), whether the analysis concerned LPS-activated splenocytes or Peyer’s patches (Fig 5C and 5D). Alternatively, junctions to a distant κ-S region truly featuring *cis* κ-CSR also occurred, and were more frequent in the *c3’RR-KIKS* than in *KIKS* samples. This difference was dramatic when assessing ongoing *cis* κ-CSR *in vitro* by stimulating naive splenocytes, where Sμ-Sγ3 κ-CSR represented about 2.5% of all “Ig recombination events” in *c3’RR-KIKS*, while remaining undetectable in *KIKS* cells (Fig 5C and 5D**)**.

LAM-HTGTS also confirmed that *in vivo*, Sμ-Sγ3 κ-CSR within the *Igκ* locus was more frequent in *c3’RR-KIKS* than in *KIKS* mice. Although less striking than *in vitro*, the difference was still significant (5.1% *vs* 4.1% of all “Ig recombination events”, p = 0.04) (Fig 5D).

Globally, the “ISD / κ-CSR” ratio (*i*.*e*. the proportion of local *vs* distant recombination) thus decreased in all conditions where *c3’RR* was included, and this effect was most significantly visible when activating naive cells *in vitro* and thus estimating *de novo* recombination (Fig 5E**).**

Altogether, these data were in agreement with flow cytometry analyses and also confirmed AID-dependence of the process since very few junctions to the bait were found in control *c3’RR-KIKS* / *Aicda*^*-/-*^ mice and none of them featured CSR (Fig 5D**).** In addition to *cis* κ-CSR junctions internal to the *Igκ* locus (κ-Sμ to κ-Sγ3), some *trans* κ-CSR recombination events joined the κ-Sμ to acceptor S regions (Sγ3, Sγ1, Sγ2b, Sγ2a, Sε, Sα) from the endogenous *IgH* locus (Fig 5C and 5D). T*rans IgH-*CSR is known to abundantly occur between both *IgH* alleles and can account for up to 15% of all normal *IgH* CSR events [27, 28]. This figure appears even higher for κ-CSR, since more than one-third of κ-CSR junctions implicated an acceptor S region from the *IgH* locus (Fig 5C and 5D), corresponding to a *trans* κ-CSR rearrangement. This *trans/cis* κ-CSR ratio did not significantly differ in both κ-CSR models and the global stimulatory effect of the c3’RR on distant κ-CSR manifested equivalently for the prevalent *cis* junctions and the alternative *trans* pathway (Fig 5E).

**Fig 6:**
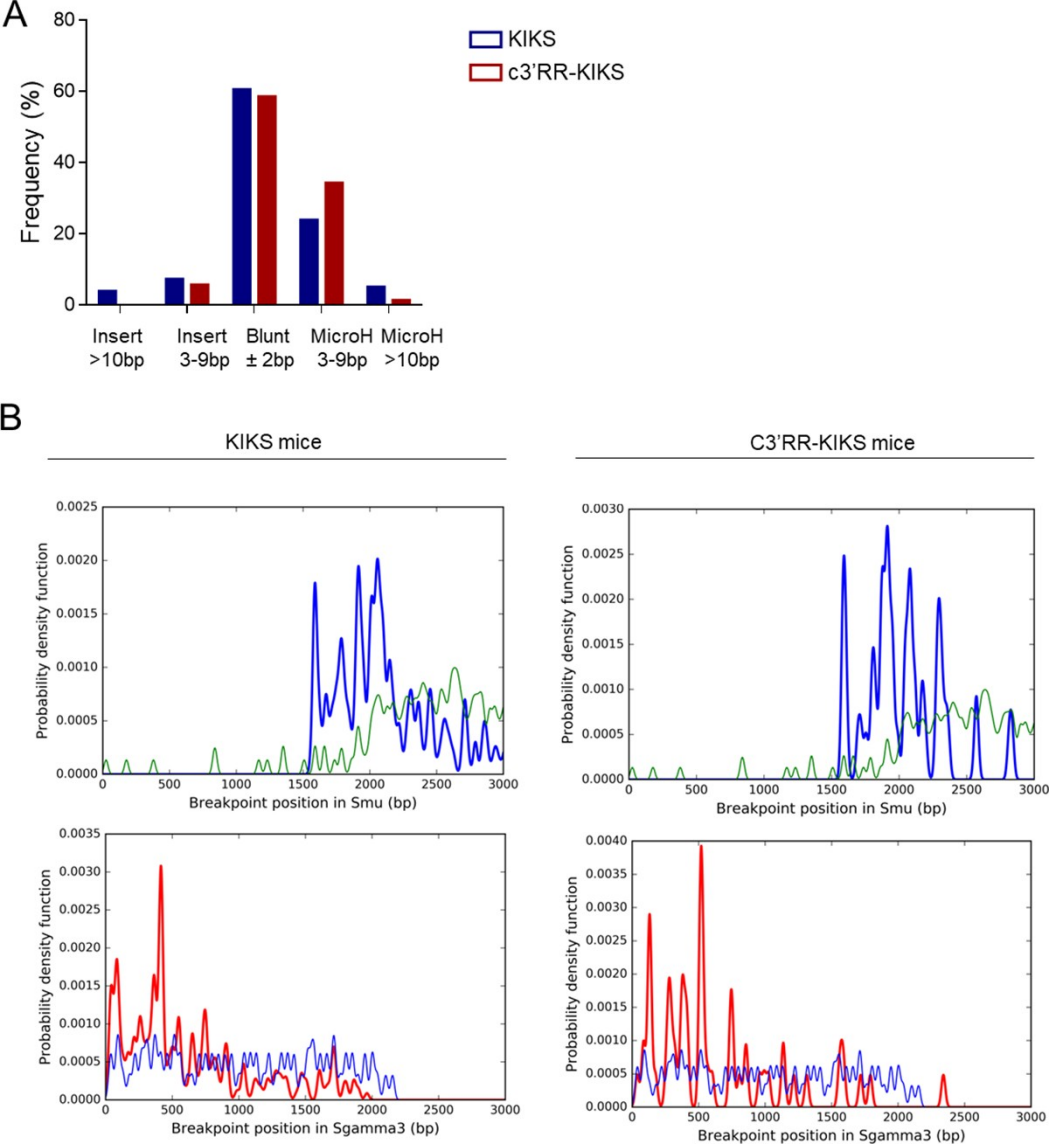
Structures and breakpoints distribution of κ-CSR junctions. (A) Structures of κ*-CSR* junctions obtained by nested-PCR from mκ^+^ cells from Peyer’s patches and analyzed using CSReport. (B) Breakpoint distribution of κ*-CSR* junctions obtained by nested-PCR from mκ^+^ cells from Peyer’s patches and analyzed using CSReport. Thick and thin lines represented breakpoints and AID motifs respectively.

Regarding repair of κ-CSR, junctions analyzed after sequencing of nested-PCR products from mCκ^+^ cells from Peyer’s patches showed predominant blunt junctions, similar to classical CSR in *wt* mice. There was thus no apparent bias in the usage of either NHEJ or microhomology-mediated repair (Fig 6A). We consistently analyzed breakpoint distribution throughout the κ-S regions and found that breaks occurred as expected around AID hot spots. No difference in breakpoint dispersion in the κ-S region occurred between the two models (Fig 6B) suggesting that c3’RR does not impact the position break but rather the outcome of the breaks.

**Fig 7:**
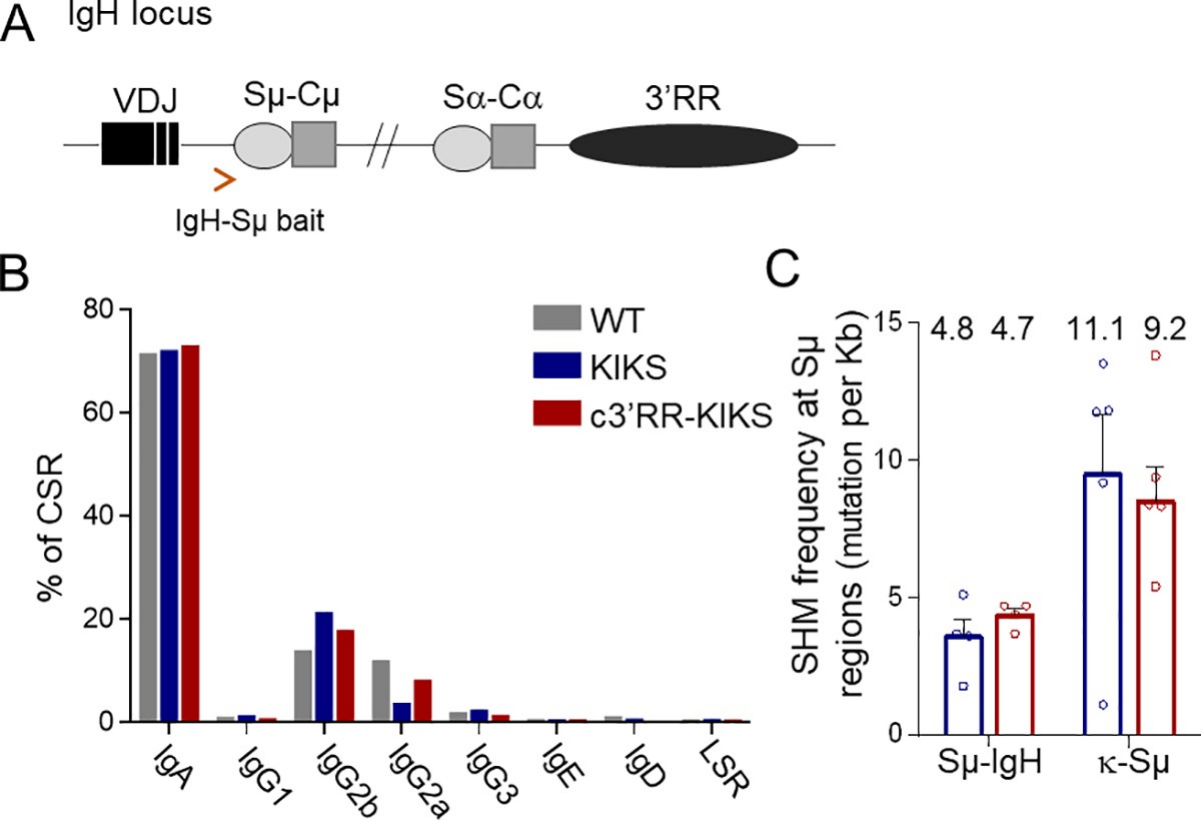
c3’RR at KI locus does not affect CSR and SHM at *IgH* locus. (A) *IgH* locus with the location of IgH-Sμ bait used for LAM-HTGS. () Percentage of switched *IgH* isotypes in Peyer’s patch B-cells from *KIKS* (n = 4), *c3’RR-KIKS* (n = 3) and *wt* (n = 3) mice obtained by LAM-HTGTS. (C) Mutation rate (mutation per kb) in IgH-*Sμ* (n = 4) and κ-*Sμ*(n = 5) after CSR recombination obtained by LAM-HTGTS from Peyer’s patches.

Globally considering κ-CSR (either in *cis* or *trans*), the present data indicate that integration of the c*3’RR* into the locus facilitated long distance synapses between AID-targeted DNA regions, rather than local deletions restricted to κ-Sμ. The *c3’RR* also tended to increase SHM of unrearranged κ-S regions (the level of which was higher in both models than observed in parallel for the *IgH* locus S regions) (Fig 4C). Strikingly, the *c3’RR*, however, facilitated the occurrence of junctions instead of less parallel local SHM. This change in the SHM/CSR ratio is in agreement with the observation made when comparing κ-CSR and parallel IgH-CSR, which can occur with minimal associated local SHM. In controls, we measured CSR at the endogenous *IgH* locus [23] and as expected, *IgH*-CSR was unaltered by the *Igκ* knock-in (with a majority of Sμ-Sα junctions in Peyer’s patches) (Fig 7A and 7B); the SHM load of these S-S junctions could thus be evaluated and was found to be 2 to 3 fold lower in the *IgH* locus (where the complete *3’RR* likely optimized the CSR/SHM ratio) compared to the *Igκ* locus (Fig 7C and S5 Table).

CSR and SHM are thoroughly intermingled processes in physiology and there are few situations where they appear uncoupled, as observed in patients with immune deficiencies and N-terminal mutations of AID, affecting SHM while preserving CSR [24, 29]. By contrast, normal *IgH* CSR usually associates with some local SHM focused on WRCY motifs even after short *in vitro* stimulation of B-cells. This minimal SHM level has long been considered as only supporting CSR in *in vitro* conditions [30]. AID targeting to S regions strongly relies on their structure, abundance of WRCY motifs, potential binding of these motifs by 14-3-3, G4-richness and ability to promote the formation of R-loops when transcribed [16, 31, 32]. This favors RNA Polymerase II stalling in association with Spt5 and recruits AID together with the exosome which locally degrades S region transcripts and exposes both strands of R-loops to cytidine deamination [33, 34]. The *3’RR* promotes DNA looping within the *IgH* locus. Such loops facilitate transcription on one hand and specific interaction of the stalled polymerase II with Spt5, AID and other cofactors, and, on the other hand, ligation of paired S regions [16, 19]. By analogy in our κ-CSR model, it seems likely that the *c3’RR* can promote an *Igκ* locus conformation that is either more susceptible to single strand lesions and SHM or forms alternative loops that favor the occurrence of DSBs, terminates SHM and supports the synapsis of S regions prior to their ligation (as during classical CSR at the *IgH* locus).

As a conclusion, using a genetically engineered *Igκ* locus as a CSR substrate, we observed that inclusion of a surrogate *3’RR* promoted CSR recombination in multiple aspects. It increased both the balance of CSR *vs* SHM on CSR-targeted S regions and the balance of recombination with distant partners *vs* local intra-Sμ deletion for the knocked-in donor Sμ region. The increased frequency of distant events primarily concerned *cis-*CSR within the modified *Igκ* locus, but also equally affected *trans-*CSR joining the *Igκ / IgH* loci.

Altogether, this study shows that the *IgH 3’RR* can instrumentally promote the transformation of AID-initiated lesions into CSR breaks rather than SHM and promote junctions with distant CSR targets rather than local deletion.

This study thus directly confirms those roles of the *3’RR* that have been previously postulated indirectly after analyzing *3’RR*-deficient mice (which show anomalies of both *IgH cis-* and *trans-*CSR). While repair pathways of junctions did not vary between control loci or loci integrating the *3’RR*, our data are consistent with the notion that the *3’RR* promotes synapsis between distant CSR targets prior to recombination. This fits with a model where the *3’RR* would participate in the assembly of a CSR factory, bringing both the loops formed in *cis* on a targeted locus, and potential other legitimate targets from other chromosomes (usually the other *IgH* allele but also here a knocked-in *Igκ KIKS* allele). This schema must thus accommodate not only the “loop model” [19] but also the data concurring to demonstrate the efficient interactions of S regions even when located on different chromosomes [27, 28, 35, 36].

That κ-CSR remains about 10 to 15-fold less frequent than *IgH* CSR in the *c3’RR-KIKS* locus is expected since the 3’ IgH super-enhancer stands as an extensive region with multiple functional modules in the *IgH* locus, which can hardly be replaced by a *c3’RR* cassette restricted to core enhancers. Notably, we previously demonstrated that the intervening sequences located in-between core enhancers architecturally contribute to the CSR-boosting effect exerted by the super-enhancer [13]. Dealing with chromosomal loops, the *IgH* locus context has by itself accumulated multiple elements favoring the occurrence of CSR loops, and the normal *IgH 3’RR* is notably followed by multiple CTCF binding elements which might ideally anchor the 3’RR within a CSR factory at the basis of a CSR loop [37]. Alt and coll thus recently proposed that CTCF and cohesin-bound elements dynamically support “loop extrusion” and enable the *3’RR* to promote secondary loops for S region synapsis and cleavage, underlining the importance of the long range locus topology and chromosomal context [38]. In an attempt to identify the necessary constituents of a functional switching domain, our comparison of two artificial Ig loci with identical structures, AID accessibility, G4-density and location of S regions within introns, but only differing by inclusion of a *c3’RR*, confirms the instrumental contribution of the *3’RR* enhancers to CSR competence beyond SHM.

## Supporting information

S1 Fig The *c3’RR-KIKS* construct was inserted into the Igκ locus by homologous recombination (not to scale). The inserted cassette includes the core Sμ, followed by the hEiκ enhancer, constant hCκ exon from the human Igκ locus and then the core *Sγ3* and *c3’RR*. A *Vκ* promoter and mutated leader exon provides transcription and splicing of *Sγ3*. The downstream floxed NeoR gene was removed by Cre-deletion to generate the germline *c3’RR-KIKS* locus. *c3’RR-KIKS* κ-CSR events join *S* regions, then excising *hCk* and yielding Ig with murine *Ck*.
(TIF)

S2 Fig Gating strategy to study B cell development in the bone marrow. (TIF)

S3 Fig A. Percentage of IgG1+ cells in spleens from NI or SRBC immunized mice (day 8) (KIKS, c3’RR-KIKS and c3’RR-KIKS / Aicda-/- mice). B. Representative dot plots of mκ and hκ staining in CD43 negative splenic B-cells. C Comparison of κ-CSR efficiency as evaluated by counting mCκ+ spleen B-cells (gated on B220+/IgM+ cells) by flow cytometry, in spleens from non-immunized (NI) or SRBC immunized (day 8) mice and from Peyers’ patches from KIKS, c3’RR-KIKS and c3’RR-KIKS / Aicda-/- mice. Percentages +/- SEM.
(TIF)

S1 Table Antibodies used in this study. (XLSX)

S2 Table Each individual experiment of κ-CSR efficiency as evaluated by counting mCκ^+^ spleen B-cells (gated on B220^+^/GL7^+^ cells) and by B220 positive cells or on B220^+^/GL7^+^ in Peyer’s patches from *KIKS*, *c3’RR-KIKS* and *c3’RR-KIKS / Aicda*^*-/-*^ mice. B: Each individual experiment of κ-CSR efficiency as evaluated by counting mCκ^+^ in *in vitro* stimulated (LPS) B-cells (gated on B220^+^).
(XLSX)

S3 Table Cycle numbers of the qPCR reaction obtained for GAPDH, κ-Sμ and κ-Sγ3 region transcripts analyzed in Peyer’s patch GC B-cells (GC) from *KIKS* and *c3’RR-KIKS* mice. (XLSX)

S4 Table Total number of mutations, total number of bp analyzed and mutation rates for [3’*Jκ5* intron—κ-*Sμ*] and κ-Sγ3 regions in *KIKS* and *c3’RR-KIKS* mice. (XLSX)

S5 Table Total number of mutations, total number of bp analyzed and mutation rates for *IgH*-Sμ or κ-Sμ regions in *KIKS* and *c3’RR-KIKS* mice. (XLSX)
