# Supplementary figures and images for: IgH 3’ regulatory region increases ectopic class switch recombination
Source: PLoS Genet. 2021 Feb 8;17(2):e1009288. doi: 10.1371/journal.pgen.1009288 (PMC7869978; doi:10.1371/journal.pgen.1009288)

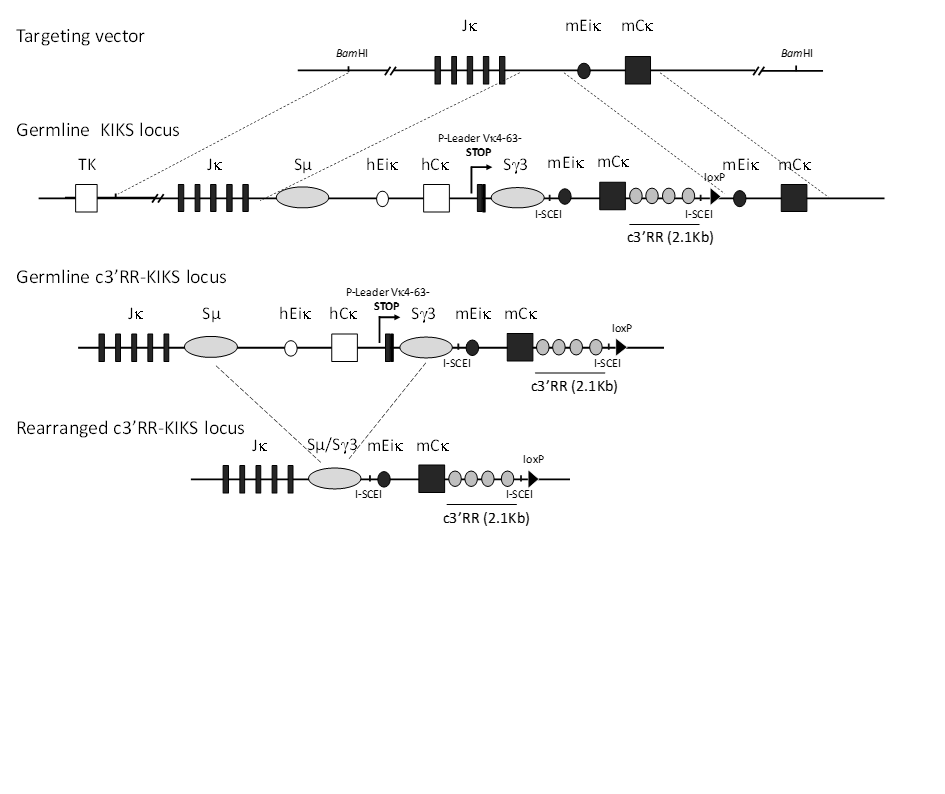

Supplement: S1 Fig — The inserted cassette includes the core Sμ, followed by the hEiκ enhancer, constant hCκ exon from the human Igκ locus and then the core Sγ3 and c3’RR. A Vκ promoter and mutated leader exon provides transcription and splicing of Sγ3. The downstream floxed NeoR gene was removed by Cre-deletion to generate the germline c3’RR-KIKS locus. c3’RR-KIKS κ-CSR events join S regions, then excising hCk and yielding Ig with murine Ck. (TIF) [file pgen.1009288.s001.TIF]

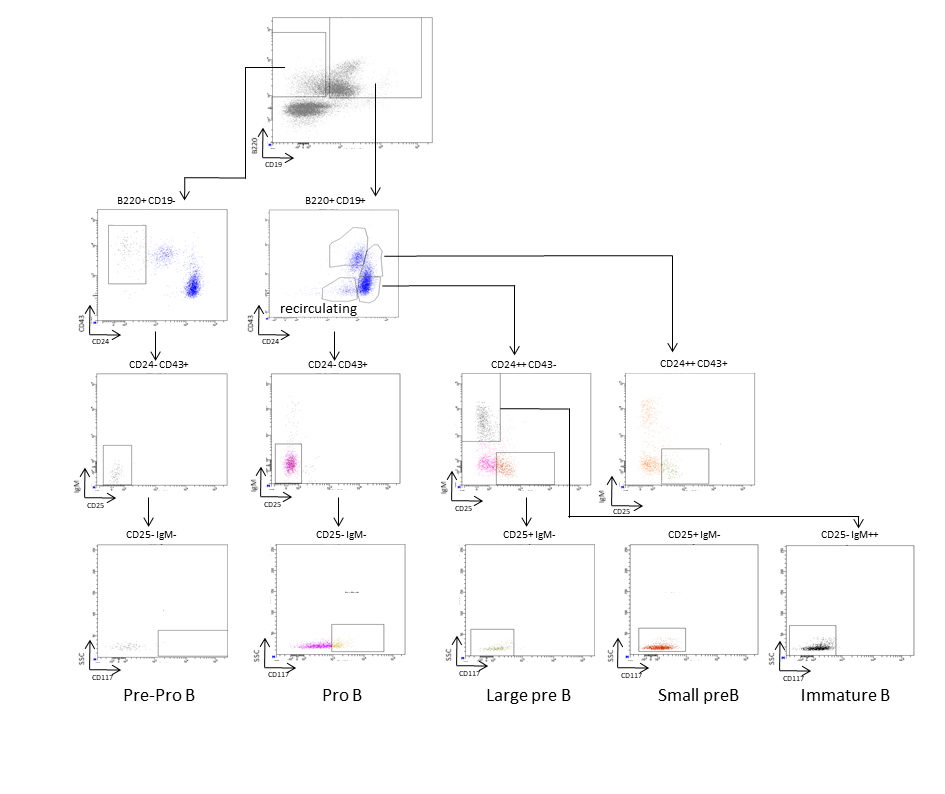

Supplement: S2 Fig — (TIF) [file pgen.1009288.s002.TIF]

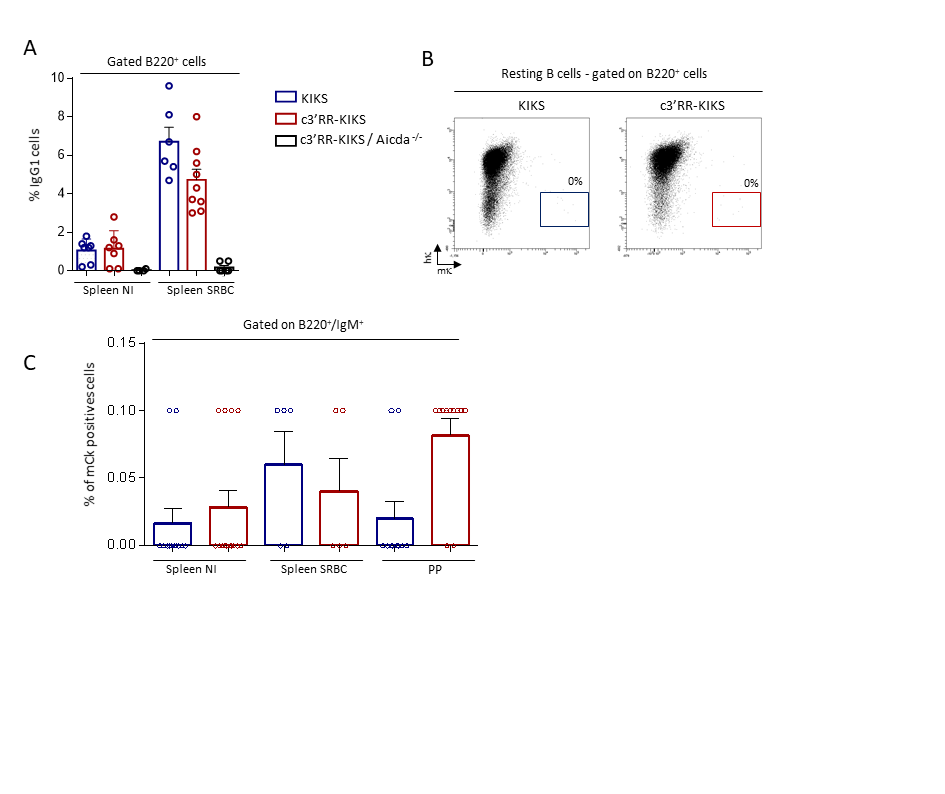

Supplement: S3 Fig — A. Percentage of IgG1+ cells in spleens from NI or SRBC immunized mice (day 8) (KIKS, c3’RR-KIKS and c3’RR-KIKS / Aicda-/- mice). B. Representative dot plots of mκ and hκ staining in CD43 negative splenic B-cells. C Comparison of κ-CSR efficiency as evaluated by counting mCκ+ spleen B-cells (gated on B220+/IgM+ cells) by flow cytometry, in spleens from non-immunized (NI) or SRBC immunized (day 8) mice and from Peyers’ patches from KIKS, c3’RR-KIKS and c3’RR-KIKS / Aicda-/- mice. Percentages +/- SEM. (TIF) [file pgen.1009288.s003.TIF]
